# Pharmacological Properties of 4′, 5, 7-Trihydroxyflavone (Apigenin) and Its Impact on Cell Signaling Pathways

**DOI:** 10.3390/molecules27134304

**Published:** 2022-07-04

**Authors:** Rameesha Abid, Shakira Ghazanfar, Arshad Farid, Samra Muhammad Sulaman, Maryam Idrees, Radwa Abdallnasser Amen, Muhammad Muzammal, Muhammad Khurram Shahzad, Mohamed Omar Mohamed, Alaa Ashraf Khaled, Waqas Safir, Ifra Ghori, Abdelbaset Mohamed Elasbali, Bandar Alharbi

**Affiliations:** 1 Department of Biotechnology, University of Sialkot, Sialkot 51310, Pakistan; 2 National Institute for Genomics and Advanced Biotechnology (NIGAB), National Agricultural Research Center, Islamabad 44100, Pakistan; shakira_akmal@yahoo.com (S.G.); midrees.omer@gmail.com (M.I.); 3 Gomal Center of Biochemistry and Biotechnology, Gomal University, Dera Ismail Khan 29050, Pakistan; mustafamuzammal1@yahoo.com; 4 Biotechnology Department, University of Gujrat, Punjab 50700, Pakistan; samrasalman2020@gmail.com; 5 Department of Microbiology, Quaid-i-Azam University, Islamabad 45320, Pakistan; 6 Biotechnology Department, Faculty of Science, Cairo University, Cairo 424010, Egypt; radwaabdallnasser@gmail.com; 7 Biotechnology and Bioinformatics Department, International Islamic University, Islamabad 44100, Pakistan; qayyummughal33@gmail.com; 8 Faculty of Agriculture, Ain Shams University, Cairo 424010, Egypt; mohamedomar@agr.asu.edu.eg; 9 Faculty of Agriculture, Benha University, Benha 13511, Egypt; alaa194229@fagr.bu.edu.eg; 10 College of Life Science and Technology, Xinjiang University, Urumqi 830046, China; waqaskustodian@gmail.com; 11 Department of Biotechnology, Fatima Jinnah Women University, Rawalpindi 46000, Pakistan; ifraghori@fjwu.edu.pk; 12 Department of Clinical Laboratory Science, College of Applied Sciences-Qurayyat, Jouf University, Sakaka 72388, Saudi Arabia; 13 Department of Medical Laboratory, College of Applied Medical Science, University of Hail, Hail 81481, Saudi Arabia; b.alharbi@uoh.edu.sa

**Keywords:** apigenin, flavonoid, apoptosis, ROS, signaling cascades

## Abstract

Plant bioactive compounds, particularly apigenin, have therapeutic potential and functional activities that aid in the prevention of infectious diseases in many mammalian bodies and promote tumor growth inhibition. Apigenin is a flavonoid with low toxicities and numerous bioactive properties due to which it has been considered as a traditional medicine for decades. Apigenin shows synergistic effects in combined treatment with sorafenib in the HepG2 human cell line (HCC) in less time and statistically reduces the viability of tumor cells, migration, gene expression and apoptosis. The combination of anti-cancerous drugs with apigenin has shown health promoting potential against various cancers. It can prevent cell mobility, maintain the cell cycle and stimulate the immune system. Apigenin also suppresses mTOR activity and raises the UVB-induced phagocytosis and reduces the cancerous cell proliferation and growth. It also has a high safety threshold, and active (anti-cancer) doses can be gained by consuming a vegetable and apigenin rich diet. Apigenin also boosted autophagosome formation, decreased cell proliferation and activated autophagy by preventing the activity of the PI3K pathway, specifically in HepG2 cells. This paper provides an updated overview of apigenin’s beneficial anti-inflammatory, antibacterial, antiviral, and anticancer effects, making it a step in the right direction for therapeutics. This study also critically analyzed the effect of apigenin on cancer cell signaling pathways including the PI3K/AKT/MTOR, JAK/STAT, NF-κB and ERK/MAPK pathways.

## 1. Introduction

Different bioactive compounds of plants have manifested many functional activities that could be an astonishing factor in the prevention of various diseases. Flavonoids are a large group of natural polyphenols which include isoflavonoids, anthocyanidins, flavonols, flavanones and flavonols. They are a class of polyphenolic compounds and are distinguished by broad biological activities that occur in many mammalian bodies, including inhibition of tumor growth [1]. Among all of the polyphenols, the five most common plant polyphenols are kaempferol, quercetin, myricetin, kaempferol, apigenin, and luteolin, which are abundantly found in over 6000 different plants located in Western Asia, Europe, Australia and Britain [2]. The molecular formula of apigenin is C_15_H_10_O_5_. It is a trihydroxyflavone that is substituted by OH functional group at the 4’, 5 and 7 position. It has reduced water solubility and greater permeability due to which it can easily pass through the plasma membrane of the host body. Moreover, it is lipophilic in nature and can survive in the gastrointestinal tract of the host under low acidic environment [3]. Apigenin is one of the most common monomeric flavonoids found in chamomile (*Matricaria recutita*), some green vegetables, artichokes, celery, parsley, herbs, sorghum and dried oregano. Chamomile is traditionally used to treat insomnia and anxiety. It shows a wide range of beneficial bioactivities in combating different diseases including cancer [4,5]. It also helps to induce autophagy in leukemia cells and acts as an antineoplastic agent.

A medical study conducted simultaneously among healthy and ovarian cancer affected women revealed that flavonoid intake has no evident connection with the cause of ovarian cancer. However, a significant decrease in the risk and potential of ovarian cancer was observed due to regular apigenin intake. After a course of medical experiments, it has been inferred that apigenin together with luteolin works as a potent chemotherapeutic agent [6].

Recently, the scientific community has explored the most promising health benefits of apigenin [7]. Apigenin has the potential to cause less intrinsic cytotoxicity and showed arrestive effects on cancerous cells as compared to other conventional flavonoids [8]. Studies have suggested that apigenin has a strong background in the prevention of numerous infections by using different experimental analyses [5]. The anti-inflammatory and antioxidant potential of apigenin [9,10] induces apoptosis and proliferation at a cellular level in host organisms and halts the overexpression of cancer-causing genes [11,12]. Apigenin regulates cell death by activating various intrinsic apoptotic pathways and caspase-3 activity, resulting in forming apoptotic protease activating factor 1(APAF) by liberating one of the components of the electron transport chain named cytochrome C [13]. This flavonoid is thought to induce antioxidant potential by activating different signaling cascades and inhibiting the activity of the NF-κB pathway. To enhance the antioxidant potential, apigenin activates antioxidant enzymes including erythrocyte superoxide dismutase, catalase, phase II detoxification enzymes and Glutathione Peroxidase (GSH-synthase) [14,15]. Apigenin not only reduced the expression of different tumor-causing genes such as TNF-α, IL-6 and CD40, but also increased the activity of the tumor-suppressing STAT1 gene via IFN-γ gene inhibition [16].

Research on mice demonstrated that apigenin reduces cholesterol levels, fatty acid production levels and obesity in mice. It regulates nicotinamide adenine dinucleotide levels and stabilizes glucose levels in the human body [17,18]. It decreases inflammation and pain by inhibiting different cellular processes and pro-inflammatory pathways. It decreases the activity and signaling of NF-κB [19], JAK2 and STAT3 [20] pathways and suppresses the expression of different cytokines [21] including Th2 cytokines, IL-4, IL-10 [22], interleukin-1β and NLRP3 genes [23]. Apigenin shows hypoglycemic effects. Its bioactive compounds can reduce insulin levels and helps to maintain the normal glucose levels in the body. Apigenin also has the potential to enhance wound and bone healing effects [24]. It is an important nutraceutical plant compound that has a noticeable effect on different chronic diseases such as Alzheimer’s, stroke, diabetes, cancer, anxiety and depression; it also has anti-microbial, anti-tumorigenic, anti-mutagenic and antiviral properties [25,26]. Recently, the scientific community has noticed the growing challenge of antimicrobial resistance (AMR) to the world. The number of multidrug-resistant organisms are increasing due to the inappropriate use of antibiotics. Although international healthcare authorities are trying to create awareness campaigns through antibiotic stewardship programs as, the risk of AMR and associated comorbidities are constantly developing. To overcome this problem, medicinal plants, especially apigenin and its bioactive compounds, can be regarded as an alternative way to treat different metabolic diseases. Literature describing the medicinal potential of apigenin has been published previously. However, an extensive review is needed to evaluate the medicinal importance of apigenin through different meta-analyses. The main objective of this review paper is to highlight the biological and pharmacological properties of apigenin which can be used to completely eradicate and obstruct the pathogenicity of various diseases. In this review, we also investigated the most promising health benefits of apigenin and its role in the cancer signaling pathways.

## 2. Anti-Inflammatory and Antioxidant Potential of Apigenin

**Figure 1:**
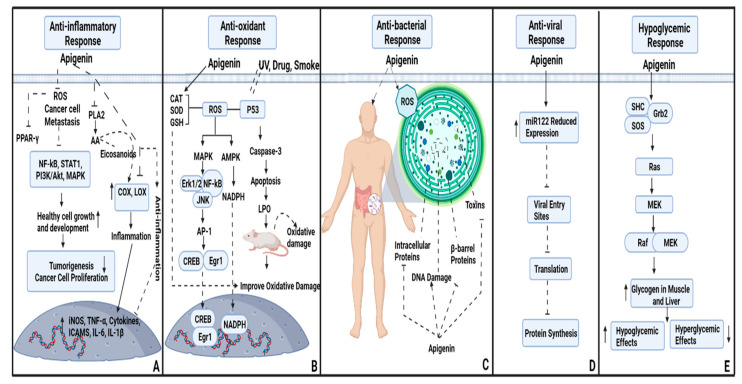
Detailed mechanism of the pharmacological properties of apigenin. (**A**) Anti-inflammatory response; apigenin inhibits the activity of reactive oxygen species and cancer cell metastasis which further inhibits several cell signaling pathways. It promotes the growth and development of healthy cells and downregulates the expression of cancer cell metastasis. Moreover, it blocks the activity of the phospholipase A2 (PLA2) enzyme which releases from the plasma membrane. Generally, PLA2 induces arachidonic acid (AA), which is a precursor of eicosanoid. Apigenin inhibits the expression of AA and promotes anti-inflammatory response in the host body. (**B**) Anti-oxidant response; host body modulates the expression of ROS when exposed to external stimuli (UV, drugs and smoke, etc.) which stimulates P53 activity and promotes apoptosis and lipid peroxidation in the host (mice) body. Apigenin consumption can significantly improve the oxidative damage caused by enhanced apoptosis and lipid peroxidation. (**C**) Anti-bacterial response; apigenin induces ROS in the bacterial cell and causes DNA damage and disruption in its cell wall. Furthermore, it can block beta-barrel proteins, intracellular proteins and toxins which are released from the bacterial cell. (**D**) Anti-viral response; apigenin enhances the reduced expression of miR122 and blocks the viral entry sites, which ultimately prevents viral DNA translation and protein synthesis. (**E**) Hypoglycemic response; apigenin stimulates Ras and MEK activity and increased the production of glycogen in the muscle and liver of the host body.

Numerous studies have shown that apigenin has gained the spotlight of the scientific community because of its anti-inflammatory and antioxidant properties. The antioxidant properties of apigenin are mediated via antioxidant enzymes including superoxide dismutases (SOD), glutathione reductase (GR) and catalase (CAT) (Figure 1) [27]. Parsley having 3.73–4.49 mg of apigenin, consumed by humans for fifteen days, resulted in elevated levels of glutathione reductase and superoxide dismutases as compared to a less flavonoid-based human diet. Another study proved that adequate apigenin doses (10, 20, and 40 mg/kg) give safety to rat livers, decrease lipid peroxidation (LPO) and secrete blood serum enzyme markers via lactate dehydrogenase, alkaline phosphatase, alanine aminotransferase, aspartate transaminase and protect rats from oxidative and membrane protein damage [28,29]. This flavonoid can suppress the activity of different diseases including cancer, those of the cardiovascular system, and diabetes by acting as a therapeutic agent, and helps to promote host health [30,31]. Apigenin has the potency to produce an enzyme-inhibitor complex and block the inflammatory activities of different enzymes [32]. A study on mouse white blood cells demonstrated that apigenin inhibits the lipopolysaccharide-induced COX-2 and NOS inhibitors activity, resulting in an anti-inflammatory response in their bodies [32] (Figure 1). Multiple cytokines, monocyte inflammatory protein (MIP-1α), intracellular cell adhesion molecules (ICAMS), and monocyte chemotactic protein (MCP-1α) also cause inflammation in the host body. Apigenin is responsible for down-regulating the expression of these cytokines via extracellular signal-regulated kinases (ERK) and mitogen-activated protein kinase (MAPK) (Figure 1) [33]. Reactive oxygen species (ROS) like alkoxyl, hydroxyl, peroxyl, nitric acid and superoxide can attack the biological molecules, resulting in oxidative damage in the human body [34,35]. These ROS help to activate multiple transcription factors like PPAR-γ, HIF-1α, Sp-1, STAT-3, AP-1 and Nrf2, and enhance different genes including inflammatory cytokines and various chronic diseases mediated through increases in their expression levels [36,37] (Figure 1). Apigenin can also treat these chronic diseases by suppressing the expression of different genes [29]. In human peripheral blood lymphocytes (HPBL), apigenin not only reduces the probability of apoptosis and mitochondrial membrane potential depolarization (ΔΨm), but also stabilizes the antioxidant status of the cell and protects the cell from oxidative damage and lipid peroxidation [38] (Figure 1).

## 3. Antibacterial Potential of Apigenin

Generally, phytochemicals like apigenin have noticeable activity against microbial and bacterial infections [26]. Many studies have investigated the potential role of apigenin against microbes. It is effective in treating amnesia and various other disorders and has antioxidant, anticancer and anti-inflammatory characteristics.

Flavones fight against bacterial infections through a fascinating mechanism. They inhibits the nucleic acid synthetization and the functionality of the cytoplasmic membrane by forming beta-barrel proteins and biofilms in bacterial species. They also interact with pivotal enzymes. When it comes to apigenin it inhibits the DNA (deoxyribonucleic acid) and biofilm formation of gram-negative *Escherichia coli* bacteria (Figure 1). Studies have shown that the antibacterial potential of flavones and phytochemicals is dependent on the selected strains of bacteria. It is worth noting that the relationship between the structural and antimicrobial activity of flavones is a subject of focus for future research [28].

Some studies have mentioned that apigenin is strongly recommended for the treatment of oral bacteria, as it possesses significant antibacterial properties. The effectiveness of apigenin with antibiotics against these bacteria increased gradually as compared to the antibiotics treatment alone, so there is a synergic effect between apigenin and antibiotics [29]. Most of these studies and experiments have been done on strain-specific bacteria in aerobic conditions, and it has been observed that apigenin can inhibit bacterial colonies and decrease their toxicity [30]. A study claimed that apigenin is abundantly present in the leaves of *Portulaca oleracea*. This plant is known because of its antioxidant, antitoxic, antimicrobial and hypoglycemic activities, which produce beneficial effects on the host body.

Apigenin can be administered in the form of tablets, capsules and oral suspensions [31,32,33,34]. Research has suggested that the most significant antibacterial activity of apigenin has been seen against gram-negative *Proteus mirabilis* [35]. Apigenin has the potential to inhibit cell proliferation via the P13k/AKT cell signaling pathway [36,37]. It shows antibacterial activities against various gram-negative and positive bacterial species [32,33,34,38].

## 4. Antiviral Beneficiary Effects

As a result of the ubiquitous spread of viral infections with no effective treatment and the continuous arrival of resistant viral strains, the improvement in the health care systems and to treat infectious diseases with novel agents is the exigency of the time. It is necessary to standardize effective and functional medications to control future outbreaks [39]. Flavonoids are effective against various DNA and RNA enveloped and non-enveloped viruses. They can prevent viruses from attaching to cells and infecting other cells by interacting with different viral replication stages and also inhibit the process of protein synthesis. Flavonoids have been discovered to inhibit viruses in a variety of ways [40].

Apigenin is a non-virulent and non-hazardous active flavonoid [41]. Studies have shown that the reduced expression of mature miR122 by apigenin consumption results in the prevention of infection caused by hepatitis C and foot and mouth disease virus at the post-entry stage (Figure 1). Reactivation of Epstein-Barr virus (EBV) and the development of virions by EBV-positive neural progenitor cells (NPC) are both inhibited by apigenin [42]. The active fraction (Fc) of *Sambucus gaudichaudiana* having apigenin as a major component could exhibit antiviral activity against poliovirus within 4 h [39,43]. Antiviral activities of apigenin were reported against the hepatitis C virus (HCV) by host factor modulation resulting in the reduction of the production of miR122, which facilitates the spread of HCV infection in vitro. Apigenin has also been used for the screening of antiviral activity against the newly developed enterovirus (EV-A71, Fuyang and BrCr strains). It inhibits enterovirus infection by blocking the virus entry sites (Figure 1). It can also disturb the interference between the viral genome with the heterogeneous nuclear ribonucleoprotein A1 and A2 (hnRNP) [44].

Furthermore, apigenin can also suppress cellular apoptosis through the cleavage of caspase-3, which is thought to be a key step in the release of viral progeny. It is also recognized as an ROS scavenger and can reduce the infection caused by ROS in EV-A71 damaged cells. Moreover, it can reduce infection-related cytokine levels. Apigenin was found to decrease the activity of the C and N junction kinase pathway, which is necessary for enterovirus replication. Despite the antiviral inhibitory activity of apigenin, some viruses including Coxsackie virus A16 (CAV16) are unaffected by apigenin consumption. EV71 is a member of the Picornaviridae and belongs to the enterovirus genus. It causes hand, foot and mouth disease (HFMD). High apigenin consumption may inhibit the replication of EV71 and EV71-mediated cytotoxic effects. The expression of viral polyproteins and the up-regulation of several cytokine-related pathways are inhibited by apigenin intake [45]. Studies revealed that the major ingredient of *Ocimum basilicum* (commonly called sweet basil) is apigenin, which shows antiviral potential [46].

In domestic pigs, the African swine fever virus (ASFV) can cause severe illness. In vitro analysis showed that apigenin has a dose-dependent anti-ASFV activity. Apigenin given to vero cells for one hour after infection reduced ASFV by more than 3 logs. Apigenin can also hinder the synthesis of ASFV-associated proteins as well as the formation of viral factories. Continuous apigenin therapy can reduce cytopathic effects in ASFV-infected cells [47]. Downstream inhibition of MAP kinase signal transduction is facilitated by apigenin consumption which ultimately prevents viral infection. Apigenin has strong antiviral activity against the C4 strain of the enterovirus genotype. At a 24.74 micromolar concentration, it decreases the cytopathogenic effect in human embryonic 293S cells by 50%. In addition, apigenin inhibits the translation of viral mRNA [43]. A compound named 7-O D-glucopyranose derived from the natural herb has the ability to protect T-cell lines from HIV [48,49].

## 5. Hypoglycemic Effects of Apigenin

Depending on the severity and scope of the condition, hypoglycemia can lead to unconsciousness and even death. It can produce ischemia or depolarization/repolarization-related alterations in the heart which can result in sudden cardiac arrest. Impaired cognitive behavior, especially in young children, causes long-term and potentially detrimental repercussions on their intellectual functionality [50]. A study has found that apigenin compound 6-C-(2″-O-α-l-rhamnopyranosyl)-β-l-fucopyranoside) can effectively lower the C_6_H_12_O_6_ level in the blood and activate the secretion of insulin [51] (Figure 1). Other studies have shown that the 6-C-β-l-fucopyranoside compound can increase glycogen synthesis in host muscle fibers [52] (Figure 1). Apigenin is found in celery vegetables that affect the metabolism of glucose and transmission in peripheral tissues [53]. The leaves of new *Bouldia laevis* plant have antidiabetic activity against patients having diabetes mellitus (metabolic endocrine disorder) [54]. 

Apigenin has been shown to suppress the activity of liver gluconeogenic enzymes, which increases the metabolic rate of glucose. It also regulates insulin secretion and produces a strong protective effect on injured pancreatic cells after streptozotocin (STZ) induction [55]. A case study reported in January 2021 found hypoglycemic effects of apigenin in diabetic rats. The sugar level and body weight of rats declined over days due to apigenin consumption [56]. Vitexin compounds in apigenin specifically reduced the blood sugar level and body weight of rats [57]. Apigenin reduced diabetic symptomology by suppressing the MAPK signaling activation, which in turn suppressed the caspase 3 and nitric oxide (NO) signaling pathway axis. Kaempferol (another flavonoid) has also been proven to reduce T2DM symptomology and its complications [57].

## 6. Role of Apigenin on Biological System

### 6.1. Apoptosis

Apoptosis can be regulated when promoters and inhibitors help to activate apoptotic processes, several genes, and inhibitory factors [58]. It can also be triggered when apoptotic receptors are exposed to their ligands or cytochrome C liberated into the cytosol [59].

IAP (inhibitors of apoptosis) family members have the potential to inhibit the function of specific caspases and induce apoptosis in them [60]. Due to IAP overexpression, several IAP inhibitors including IAP-1 and IAP-2 were also observed in prostate cancer cell lines [61]. Studies suggested that when Bax (pro-apoptotic protein) interacts with the C-terminus of Ku70 protein, the normal apoptotic process can be inhibited [62]. Recent studies have found that the Ku70 protein is responsible for cancer progression and metastasis; however, Ku70–Bax bioconjugate can also act as a promising therapeutic target [63]. Apigenin consumption may drastically decrease the total number of tumor cells and activate the process of apoptosis leading to a reduction in Bcl-xL and Bcl-2 levels and activating the Bax protein. The expression of HDAC1 gene and class I histone deacetylases can also be inhibited by apigenin therapy, which causes apoptosis in a cancer cell. Apigenin also decreased HDAC1 occupancy at the XIAP promoter, implying that histone deacetylation is essential for XIAP downregulation. These findings have confirmed the apoptotic behavior of apigenin in prostate cancer cells [64].

Apoptosis-associated proteins in apoptotic cells have been the subject of discussion in many studies. Studies have suggested that apigenin can be treated against various types of cancers [8]. Apigenin causes apoptosis more efficiently in blood cancer cells as compared to other cancer cells [65] however, the anticancer activity of apigenin in the cholangiocarcinoma cell lines has not been discovered yet [66]. Apigenin blocks the PI3K/AKT pathway, and induces several caspases and cell cycle arrest in different cancer types [67].

Apigenin can also inhibit AKT signal transduction. AKT is an anti-apoptotic signaling molecule that can mediate PI3K-dependent cell survival responses [68]. Bcl-2 proteins are significantly involved in apoptosis. The mitochondrial apoptotic pathway is activated because of the changes that occurred in the pro- and anti-apoptotic behavior of these proteins [69]. Apigenin treatment induced apoptosis in T24 cells and suppressed AKT phosphorylation in a dose-dependent manner and ultimately blocked the PI3K/AKT pathway, which inhibits cancer proliferation.

A study reported that apigenin treatment on the HeLa cell lines can remarkably enhance the activity of p21/WAF1 protein. The transcriptional upregulation of this protein is p53-dependent. Apigenin also enhances the activity of the Fas/APO-1 and caspase-3 proteins, and both proteins are directly linked to apoptosis. The results of this study showed that apigenin can act as a cervical cancer inhibitory agent [70]. Apigenin shows synergistic effects in combined treatment with sorafenib in the HepG2 human cell line (HCC) in less time and statistically reduces the viability of tumor cells, migration, gene expression and apoptosis. Anti-cancerous drugs are effective but they also harm living bodies due to their toxicities. A combination of these anti-cancerous drugs with apigenin has shown promising potential against tumors. RT-PCR results demonstrated that the efficacy of apigenin showed a notable rise in the expression of p21 protein in the 50 uM 4, 5, 7-Trihydroxyflavone treated group, which indicated that apigenin has strong anti-cancer activity on human cancer cells [71]. Apigenin can induce autophagy and apoptosis, which inhibits cancer metastasis. It can prevent cell mobility, maintain the cell cycle, and stimulate the immune system [72]. In a study, apigenin-treated human lung cancer H460 cell lines were morphologically analyzed at different times to check the pro-apoptotic activity. Results suggested that apigenin can promote apoptosis in H460 cells by decreasing the membrane potential of the mitochondrial-dependent pathway, increasing the expression of ROS and calcium accumulation in lung cancer [73]. This bioactive compound can effectively work against human breast cancer cell lines T47D and MDA-MB-231. The mortality of both cell lines was attributed to apoptosis, high levels of caspase, poly (ADP-ribose) polymerase PARP-cleavage, and a high Bax/Bcl ratio [74]. Furthermore, apigenin-treated cells inhibited autophagy. Such combinations of autophagy induced apoptosis and provided significant potential to combat human breast cancer. It is hypothesized that apigenin consumption promotes apoptosis and activates extrinsic and intrinsic apoptotic pathways. A study reported the potency of apigenin in human osteosarcoma by inducing apoptosis in a human bone osteosarcoma epithelial cell line, (U-2OS) and it also suppresses the xenograft tumor growth [75].

### 6.2. Immune Response

Plant secondary metabolites (such as alkaloids, phenolics and terpenes) have known medicinal properties including the antioxidant, antidiabetic, antimicrobial, antimutagenic, anti-inflammatory and anti-clotting [76]. Studies have shown that apigenin intake in human hosts significantly fluctuates the immune system response. The immune cells have a specified protein named programmed cell death 1 (PD1); its receptors are PD-L1 (PD-Ligand1) and PD-L2 (PD-Ligand2). These receptors are present on the surface of either macrophages or on the dendritic cells [77]. When PD1 comes in contact with its receptor, the output would be in the form of activation of a PD1 signaling pathway. Several autoimmune diseases/inflammation may occur if PD1/PD-L1 activates the immune system at an inappropriate time [78]. Pardoll et al. reported that PD-L1 complex can be expressed in cancerous cells, leading to antigenic escape in them [79].

Apigenin can stimulate the expression of the NF-κB signaling cascade, which enhances the level of WBCs in the human body [80]. Another study on the rat model reported that consumption of soluble apigenin (Apigenin K) may not only lower the inflammatory and colonic damage level in rats but also stabilize the activity of TNF-α, IL-6 and chemokine ligands. Li et al. suggested that apigenin intake can also suppress ovalbumin (OVA) activity in Th17 cells and ameliorates asthmatic progression in the white blood cells of mice [81].

Cancerous cells can also cause immunity damage in the host body by the inhibition of T effector cell expression and also enhance the levels of T regulatory cells. Research on the pancreatic mouse model reported that apigenin effectively enhanced the activity of T cells and downregulated the activity of T regulatory cells, which increased the survival rate in mice [82]. Continuous consumption of apigenin for two weeks could decrease the level of IgE antibodies, while no apparent effect has been shown on IgG, IgM and IgA antibodies levels [83].

### 6.3. Autophagy

Autophagy is associated with lipid breakdown and helps in the metabolism of lipid droplets (LDs). However, no investigation has been done to evaluate apigenin’s lipid-lowering mechanism in the context of autophagy. Apigenin has previously been found to have anti-adipogenic effects in HepG2 cells. Apigenin’s role in autophagy and lipid accumulation in palmitic acid (PA)-induced HepG2 cells has also been documented in the literature. Apigenin increased autophagosome formation and the cytosolic light chain ratio (LC3-II/I) in HepG2 cells. In PA-treated HepG2 cells, apigenin promoted autophagy and enhanced autophagic lipid breakdown [84,85]. Apigenin helped to increase the metabolism of lipids by inducing the AMPK-SREBP pathway in HepG2 cells. In addition, apigenin decreased cell proliferation and activated autophagy by inhibiting different cellular pathways in HepG2 cells. *p*-mTOR, P62, and LC3 expression in PA-treated HepG2 cells were investigated by western blotting. These cells were treated with apigenin and PA at various concentrations over a variety of periods, and it was observed that the cytosolic light chain ratio in apigenin exposed cells increased after every 3 h [86].

Apigenin therapy may alleviate the restricted autophagic flow and lipid buildup caused by PA through a new mechanism. Furthermore, it was demonstrated that the autophagic mechanism is critical for lipid breakdown. Both PA and apigenin increased autophagosome accumulation, and the cytosolic light chain ratio was enhanced even more. A recent study discovered that hepatic steatosis occurred due to the deficiency of autophagic flux in the host body [87]. Excessive lipid aggregation has been demonstrated to hinder autophagy by reducing lysosomal acidification and the activation of different hydrolyses in cells. It is worth noting that conventional medicines such as metformin are useful for the treatment of metabolic illnesses, which helps to reduce the hepatic fat levels via autophagy [88].

Similarly, according to a prior study, epigallocatechin gallate and kaempferol improved the breakdown of LDs by facilitating autophagic flow [89]. According to a lactate dehydrogenase (LDH) release test, apigenin administration promoted cell mortality in primary human epidermal keratinocytes (HEKs) and the cutaneous squamous cell carcinoma cell line COLO-16. Apigenin therapy, on the other hand, restored autophagy inhibition in ultraviolet B treated HEKs. Furthermore, apigenin consumption reversed the ultraviolet B radiation-induced downregulation of the ataxia-telangiectasia and the unfolded protein response (UPR) regulatory proteins in HEK cells. UVB-induced apoptosis and cell death in HEKs were likewise reduced by apigenin administration. It was demonstrated that apigenin has a unique effect in UVB-damaged keratinocytes, implying that it could be used as a photoprotective agent [90]. It can cause apoptosis in PEL cells and reduce the level of intracellular ROS [91].

Apigenin treatment for two weeks dramatically reduced mTOR activity. The results of apigenin administration against ULK1 and AMPK pathways were opposite to that of mTOR signaling transduction, which regulates autophagy via mTOR/AMPK/ULK1 pathway. Apigenin has antidepressant effects in both chronic restraint stress rats and mice [92]. Autophagy is considerably impaired in the sera of people with severe depression in clinical studies. It has been linked to the development of several neurological illnesses such as Alzheimer’s disease, Parkinson’s disease as well as depression [93]. The current study highlighted some of the polyphenolic and bioactive potential of apigenin. However, more metabonomic, proteomic, and transcriptomic research is needed as the next step in developing apigenin as a therapeutic drug.

## 7. Role of Apigenin in Cell Signaling Pathways

### 7.1. PI3K/AKT/MTOR Pathway

The phosphatidylinositol 3-kinase, protein kinase B and the mammalian target of the rapamycin (PI3K/AKT/mTOR) cascade have been considered to regulate the morphological characteristics of healthy cells and promote the activity of different genes, metastasis, tumorigenesis, transcription, translation, cell proliferation, migration and cell development in cancerous cells [94,95,96]. PI3K belongs to the lipid kinase family and is divided into the following classes: class-I, class-II and class-III. class-I and class-III are heterodimers; the former has p85 and p110 subunits, while class-III has Vps 34 and Vps15/p150 subunits. Class-I is not only a part of the insulin signal transduction but also has the potential to regulate the AKT pathway, the immune response and plays a significant role in cell proliferation. Class-III is involved in intracellular trafficking and phagocytosis. Class-II PI3Ks are monomers and have a specialized C-terminal domain that typically binds with lipids instead of Ca^+2^ because of the absence of aspartic acid residues [97,98,99,100]. The central participant of PI3K/AKT/MTOR is AKT, which is activated by the phosphorylation of PI3K. It can trigger ribosomes for protein synthesis and helps in the suppression of TSC1/2 complex and other cell cycle inhibitors, resulting in the activation of the mTOR pathway [101]. mTOR also belongs to the kinase family, having a molecular weight of about 289 kDa. It helps in phagocytosis, synthesis, transcription and translation. Studies have shown that this transduction signaling cascade is linked with cancer progression [102,103].

**Figure 2:**
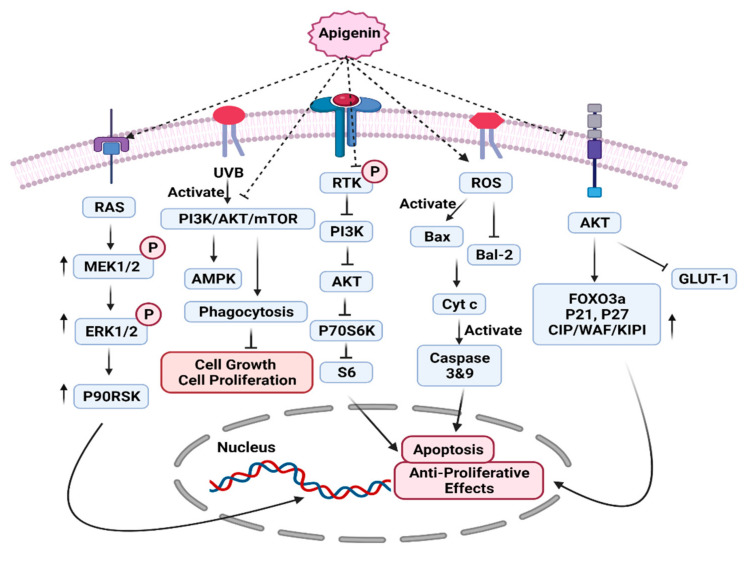
Modulation of PI3K/AKT/MTOR pathway by apigein.

On the activation of this pathway, several of its proteins are regulated by the phosphorylation of the AKT molecule, which is responsible for cell growth and survival. Apigenin consumption can significantly halt the ATP binding site of phosphatidylinositol 3-kinase, thereby ultimately reducing the functionality of AKT [104,105] (Figure 2). The expression of a transcription factor named FOXO3a (Forkhead box O3) is enhanced by apigenin in breast cancer patients, which downregulates AKT signal transduction. The survival of breast cancer cells is inhibited by upregulating the p21, CIP/WAF, KIPI, and p27 inhibitors [106,107] (Figure 2). Researchers have reported that apigenin consumption has the potential to inhibit P70S6K and S6 proteins, particularly in JAR and JEG3 cells, and to upregulate the expression of ERK1/2 as well as P90RSK proteins. Under specific conditions and cancer types, apigenin consumption can also upregulate the expression of ERK1/2 as well as P90RSK. Some inhibitors like LY294002 and U0126 directly inhibit the expression of PI3K and ERK1/2 [107].

Apigenin also has the potential to stabilize the blood glucose concentration in the body by lowering the activity of glucose transporter protein (GLUT-1) (Figure 2). The reduced expression of GLUT-1 is observed by the inhibition of the PI3K/AKT signaling cascade. This flavonoid helps to activate the Bax protein and inhibits the expression of Bcl2. As a result of this, cytochrome C releases, which directly activates the expression of caspase 3 and 9 and leads to apoptosis and anti-proliferative effects (Figure 2). A study on a mice model by Bridgeman et al. suggested that ultraviolet B (UVB) radiation exposure can activate PI3K/AKT/MTOR, which can cause the progression of skin cancer. Interestingly, apigenin suppressed mTOR activity, raised the UVB-induced phagocytosis and reduced cancerous cell proliferation and growth [108] (Figure 2).

Apigenin has demonstrated its efficacy in the treatment of prostate cancer, producing an inhibitor effect that aids in the cancer cell invasion and migration in a dose-dependent manner. In melanoma A375 and C8161 cell lines, 40 mg of apigenin was found to inhibit the activity of cancer cells via AKT/mTOR signal transduction [109].

### 7.2. JAK/STAT Pathway

**Figure 3:**
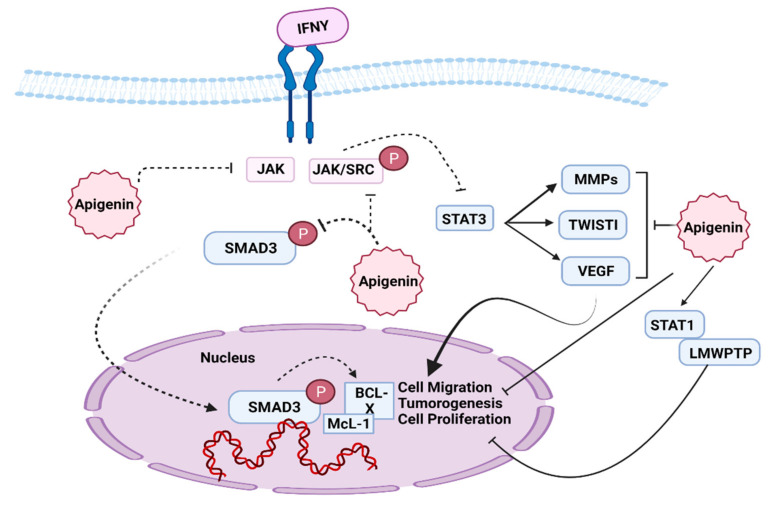
Inhibition of JAK/STAT pathway by apigenin.

JAK/STAT signaling cascade is responsible for apoptosis, cell division, cell proliferation, tumorigenesis, and arbitrates several cellular responses to cytokines [110] (Figure 3). JAK mediates its control by mediating four proteins that include JAK1, JAK2, JAK3, TYK2 and four domains: FERM, SH2, kinase and pseudokinase having almost 400, 100, 250 and 300 residues, respectively [111], while STAT contains a total of seven proteins: STAT1, STAT2, STAT3, STAT4, STAT5A, STAT5B and STAT6, which have 575–680 residues. It also has several transcriptional activation domains located at the C terminal of amino acids [112].

The JAK/STAT cellular pathway is linked with multiple cancers, and it has been investigated in various animal models [113]. The STAT protein is phosphorylated by several kinases that form dimers which are then moved to the nucleus and act there as an activation factor for transcription. The potential role of apigenin was investigated previously; it prevents the growth and development of tumor cells by inhibiting JAK/SRC phosphorylation, resulting in the suppression of STAT3 activation, which further inhibits the transference of STAT dimers to the nucleus [114] (Figure 3). Various genes are regulated by STAT3 including MMPs, TWIST1 and VEGF, and studies have suggested that these genes trigger metastasis, cell migration and tumor formation, but the expression of these genes is inhibited by apigenin treatment, which significantly blocked the activity of STAT3 [115] (Figure 3). Apigenin can also halt STAT3, STAT5 gene expression and enhance the activity of STAT1, STAT2 and LMWPTP, which inhibits cell proliferation, tumor invasion and tumorigenesis [116] (Figure 3).

### 7.3. NF-kB Pathway

The efficiency of apigenin is regulated by the repression of COX-2, iNOS, MMP-9, and cyclinD1 gene expression. All of these genes are controlled by the Nuclear Factor-kappaB (NF-kB) pathway. Apigenin is also reported to cause apoptosis in many cancer types [117] via the inhibition of DNA replication, protein kinase inhibition, ROS production, damage to mitochondria, and interference with Ku70-Bax interaction. Increased survival and proliferation are reported to be involved with NF-kB signal transduction which induces transcription of those targeted genes with their product, which can inhibit different stages of programmed cell death [118,119].

**Figure 4:**
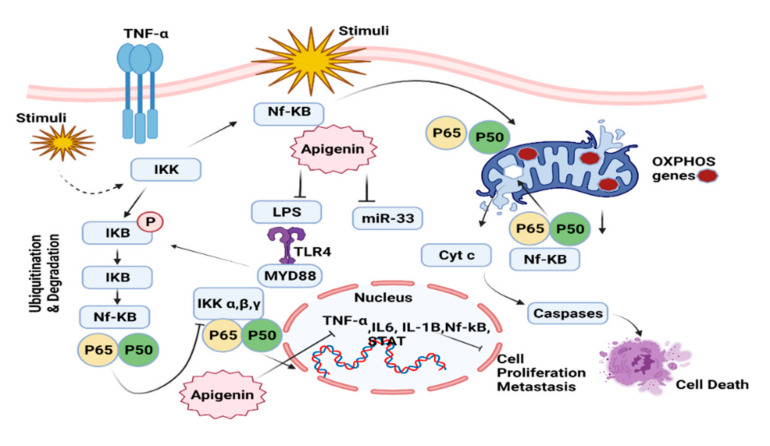
Downregulation of NF-kB pathway by apigenin.

This pathway is composed of different dimers that are made with several different segments: NF-kB1 (p50/p105); NF-B2 (p52/100); RelA (p65); RelB; and c-Rel proteins. The inhibitors of the IB (Investigator’s Brochure) family includes IB, IkB, Bcl-3, p100, and p105, which control the NF-kB proteins. NF-kB heterodimers having p65 and p50 can be found in the cytoplasm and are bound to IB subunits in their inactive form. The activation of kinase complex (IKK) phosphorylates the IB subunit at a serine residue and induces ubiquitination and proteasomal degradation, which causes localization of the nucleus and the transcriptional activation of NF-kB [120] (Figure 4).

Hematological malignancies, as well as cancer of the skin, uterine cervix, lung, esophagus, prostate and pancreas, have all been connected with abnormal NF-kB activation [121]. Researchers noted that NF-kB is stimulated constitutively in human prostate cancer tissue, prostate tumor xenografts and in the transgenic adenocarcinoma of the mouse prostate model (TRAMP), which mimics progressive types of human prostate cancer [122]. The enhanced activity of NF-kB is connected to the production of different diseases, and its nuclear localization is linked with the biochemical recurrence and poor state of prognosis [123].

In addition, the IB protein family members (IB, IB, IkB, Bcl-3, p100, and p105) control the NF-kB family members. NF-kB exists in the heterodimeric state in the cytoplasm and binds to IB in an inactive state. TNF, FasL, and TRAIL are only a few of the signal molecules that activate the IKK complex, causing IB to be phosphorylated and degraded. NF-kB then flows towards the nucleus. Pro-survival genes, cell cycle-related genes (cyclin D1), (VEGF), inflammatory cytokines, and tumorous genes are among the target genes activated by NF-kB (COX-2) [124]. Apigenin therapy prevents NF-kB activation in most cases, both in vitro and in vivo. Shukla et al. demonstrated that apigenin consumed by transgenic adenocarcinoma prostate mice prevented prostate tumorigenesis by interfering with the NF-kB signaling pathway in a prostate mouse model. Apigenin therapy can also reduce prostate tumor volume and significantly eliminate cancer cells [125]. According to the studies, the use of apigenin can inhibit the phosphorylation and degradation of IB by halting IKK activation, which suppressed the activation of NF-kB [126] (Figure 4).

Apigenin cannot affect NF-kB expression in the human non-small cell lung cancer cell lines (A549), but it can prevent the translocation of NF-kB from the cytoplasm to the nucleus, resulting in the inhibition of apoptosis-blocking target genes. Apigenin also inhibits the degradation of IB in lung cancer, preventing IB from being separated from the NF-kB heterodimer [127,128]. Apigenin therapy inhibited NF-kB nuclear translocation and AKT activation, as well as modulating MAPK signaling pathways, in malignant mesothelioma in vitro and in vivo [129,130]. It is reported that epidermal growth factor receptor (EGFR) activates the PI3K/AKT pathway, which may lead to the activation of IKK, IKBa phosphorylation on serine 32/36, NF-kb translocation and HER-2 signaling through protein kinase (Ck-2), which results in the NF-kb transactivational activity (Figure 4). Apigenin also blocks LPS and miR-33 activity and ultimately inhibits the expression of several cytokines and tumor causing genes in the nucleus. As a result of this, it can prevent cancer cell proliferation and metastasis (Figure 4).

### 7.4. ERK/MAPK Pathway

**Figure 5:**
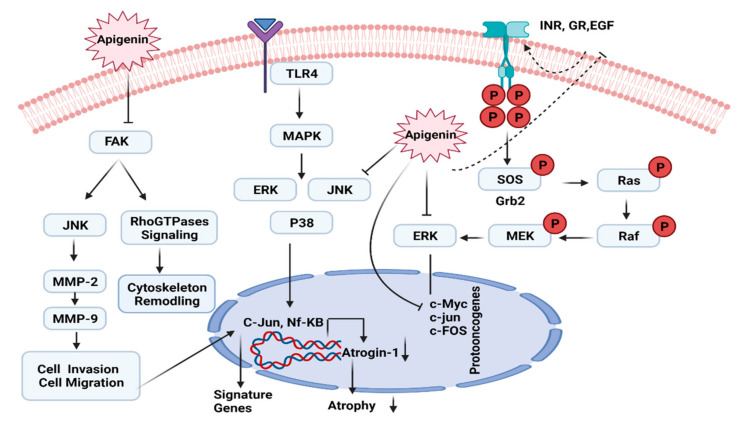
Inhibition of ERK/MAPK pathway by apigenin.

Apigenin has been shown to activate the cleaved caspase-3 and PARP expression sites, down-regulate the Twist1, *p*-mTOR, ERK1/2 proteins, deactivate FAK/ERK1/2 pathways and halt the phosphorylation of STAT3 in different melanoma cases [131,132] (Figure 5). The ERK family is organized into three different key categories which include ERK/MAPK, p38 kinase and c-Jun/SAPK. Cell development and homeostasis are dependent on these kinases. Several members of the ERK protein family take part to regulate the MAPK-ERK pathway. The proper and stable functioning of both the upstream and downstream targets of the MAPK/ERK pathway is disrupted by the overexpression of any family member of the ERK family [133]. ERK signaling is stimulated in a variety of cancers by mutations in kinase genes (Figure 5). Apigenin is thought to influence the ERK signaling cascade. It can inhibit the MAPK/ERK potential of proliferation by modulating the expression of AKT, ERK, and NF-kB [134] (Figure 5).

Apigenin can stimulate AKT and ERK inhibition because of the impact of the ABT-263 molecule, inducing apoptosis, as observed in in vitro and in vivo experiments [135] (Figure 5). It has shown the capability to trigger apoptosis in prostate cancer cells. In a prostate cancer mouse model, it directly attacks the IGF/IGFBP-3 protein and lowers its expression by reducing *p*-AKT and ERK1/2 [136].

Furthermore, apigenin is linked with the inhibition of proliferation in cutaneous melanoma cells of humans. It also inhibits ERK1/2 and FAK phosphorylation, which leads to decreased integrin synthesis [130,137] (Figure 5). A study reported that apigenin can increase the levels of ERK1/2 and inhibit the activation of p38 kinase in lung and prostate cancer cell lines. Results showed that apigenin can induce apoptosis by inhibiting the MAPK/ERK signaling pathway [138,139].

To block the AKT signaling pathway, apigenin has been shown to affect the MAPK/ERK signaling cascade in several malignancies. Apigenin substantially inhibits cell proliferation, invasion, migration, and triggered G2/M phase arrest and death in human melanoma A375 and C8161 cell lines, and reduces the activity of *p*-ERK1/2, *p*-AKT, and *p*-mTOR [140]. It can increase TRAIL-induced apoptosis in non-small cell lung cancer cells by inducing DR4/DR5, AKT, ERK, and NF-B signaling. Apigenin was suggested to improve ABT-263-induced anticancerous activity in the HCT116 and DLD1 cell lines in in vitro and in vivo by inhibiting the pro-survival regulators AKT and ERK [132]. Furthermore, in an autochthonous mouse prostate cancer model, the co-inhibition of AKT and ERK signaling was reported. Apigenin treatment successfully slowed the progression of prostate cancer in mouse models by lowering IGF/IGFBP-3 and reducing *p*-AKT and *p*-ERK1/2 expression [141]. In other investigations, this flavonoid inhibited the ERK signaling pathway as well as protein kinases including focal adhesion kinase (FAK) (Figure 5). Hasnat et al. reported that apigenin produced anoikis (programmed cell death) in human cutaneous melanoma cells by lowering integrin protein levels and suppressing FAK and ERK1/2 phosphorylation [139] (Figure 5). Apigenin also inhibited the effects of 4-(methylnitrosamino)-1-(3-pyridyl)-1-butanone on pancreatic cancer cell proliferation and their migration in the pancreatic cancerous cells by targeting the protein kinases. i.e., FAK and ERK. Shukla et al. found that the administration of apigenin raised phosphorylation of ERK1/2 and JNK1/2 while decreasing the phosphorylation of p38 in human prostate cancer LNCaP and PC-3 cells. The regulation of this flavonoid in the MAPK pathway resulted in apigenin-induced cell cycle arrest in the G0/G1 phase. Apigenin was discovered to cause the death of cancer cells and diminish cancer cell survival in human choriocarcinoma cells by inhibiting the AKT-mTOR pathway and by boosting the phosphorylation of ERK1/2 and P90RSK in an appropriate dose-dependent manner [107]. Apigenin can directly inhibit the activity of membrane receptors and suppresses the expression of protooncogenes in the nucleus. It can also block ERK and JNK activity and downregulate Atrogin-1 gene expression (Figure 5).

## 8. Possible Side Effects of Apigenin

In general, intentionally consuming higher doses of dietary flavonoids, such as apigenin, is considered safe and may even have health benefits. There is a low risk of toxicity. The amount of apigenin in an individual’s diet is highly unlikely to reach an amount that can cause harm [142].

There is a slightly higher risk of side effects when a person intentionally takes higher doses of a dietary supplement. The potential side effects of apigenin may include:

- Upset stomach
- Muscle relaxation
- Sedation

If a person experiences stomach discomfort after consuming chamomile extract, which is sometimes taken for its high levels of apigenin, then they should discontinue its use immediately. Topicals containing the nutrient may cause skin irritations in some people. A person should contact their healthcare provider before taking apigenin supplements if they take any prescription medications. There is a significant chance of drug interactions with apigenin, particularly if an individual takes cyclosporine, warfarin, or some types of chemotherapy drugs [143].

## 9. Conclusions

This review has highlighted the biological significance of apigenin and how this bioactive compound acts as a therapeutic agent because of its well-known characteristics including the anti-bacterial, anti-tumor, anti-inflammatory, anti-oxidant, antiviral and as well as hypoglycemic ones. This flavonoid can suppress the activity of different diseases including cancer, those of the cardiovascular system, and diabetes by acting as a therapeutic agent and helping to promote host health. It is hypothesized that apigenin consumption promotes apoptosis in cancer cells and activates extrinsic and intrinsic apoptotic and cancer signaling pathways. Generally, the consumption of higher doses of apigenin is considered safe and may have health benefits. However, there is a significant chance of drug interactions with apigenin, particularly if a person takes cyclosporine, warfarin, or some types of chemotherapy drugs. It is worthy of note that due to the beneficial effects of apigenin, it could be used as a dietary supplement or as a feed additive in the near future. However, more metabonomic, proteomic, and transcriptomic research is needed as the next step in developing apigenin as a therapeutic drug.

## Data Availability

Not applicable.
